# Bakterielle Zoonosen mit Bedeutung für den öffentlichen Gesundheitsschutz in Deutschland – Vorkommen, Verbreitung und Übertragungswege

**DOI:** 10.1007/s00103-023-03703-6

**Published:** 2023-05-23

**Authors:** Hendrik Wilking, Sandra Beermann, Ides Boone, Johannes Dreesman, Volker Fingerle, Jörn Gethmann, Raskit Lachmann, Marina Lamparter, Anne Mayer-Scholl, Anika Meinen, Meike Schöl, Beneditta Suwono

**Affiliations:** 1 Fachgebiet Gastrointestinale Infektionen, Zoonosen und tropische Infektionen, Abteilung für Infektionsepidemiologie, Robert Koch-Institut, Seestr. 10, 13353 Berlin, Deutschland; 2 Abteilung Öffentliche Gesundheit, Bundesministerium für Gesundheit, Berlin, Deutschland; 3 Fachgebiet Nosokomiale Infektionen, Surveillance von Antibiotikaresistenz und -verbrauch, Abteilung für Infektionsepidemiologie, Robert Koch-Institut, Berlin, Deutschland; 4 Niedersächsisches Landesgesundheitsamt, Hannover, Deutschland; 5 Bayerisches Landesamt für Gesundheit und Lebensmittelsicherheit, Nationales Referenzzentrum für Borrelien, Oberschleißheim, Deutschland; 6 Institut für Epidemiologie, Friedrich-Loeffler-Institut – Bundesforschungsinstitut für Tiergesundheit, Greifswald – Insel Riems, Deutschland; 7 Nationales Referenzlabor (NRL) für Salmonella, Bundesinstitut für Risikobewertung, Berlin, Deutschland; 8 Konsiliarlabor für Leptospiren, Bundesinstitut für Risikobewertung, Berlin, Deutschland; 9 ÖGD-Kontaktstelle | Krisenmanagement, Ausbruchsuntersuchungen und Trainingsprogramme, Abteilung für Infektionsepidemiologie, Robert Koch-Institut, Berlin, Deutschland; 10 Fachgebiet Surveillance und elektronisches Melde- und Informationssystem (DEMIS) | ÖGD Kontaktstelle, Abteilung für Infektionsepidemiologie, Robert Koch-Institut, Berlin, Deutschland

**Keywords:** One Health, Bakterielle Infektionen, Schnittstelle Mensch-Tier, Epidemiologie, Inzidenz, One Health, Bacterial infections, Human-animal interface, Epidemiology, Incidence

## Abstract

Bakterielle zoonotische Erreger sind häufig Auslöser von Erkrankungen mit teilweise schweren Verläufen. Sie sind wechselseitig zwischen Tieren (sowohl Wild- als auch Haustieren) und Menschen übertragbar. Die Transmissionswege sind sehr variabel, so kann die Übertragung u. a. durch orale Aufnahme über Lebensmittel, respiratorische Aufnahme über Tröpfchen und Aerosole sowie über Vektoren wie Zeckenstiche oder Nagerkontakte stattfinden. In diesem Zusammenhang sind auch das Auftreten und die Verbreitung von antibiotikaresistenten bakteriellen Erregern von zunehmender Bedeutung für den öffentlichen Gesundheitsschutz.

Die Ausbreitung zoonotischer Erreger wird aktuell durch zahlreiche Faktoren verstärkt. Dazu gehören die Zunahme des internationalen Warenverkehrs, die Einengung der Lebensräume von Tieren und der dadurch zunehmend engere Kontakt zwischen Menschen und Wildtieren. Aber auch eine veränderte Tierhaltung in der Landwirtschaft und Klimaveränderungen können zur Ausbreitung beitragen. Der öffentliche Gesundheitsschutz und die Erforschung von Zoonosen sind deshalb von besonderer krankheitspräventiver, aber auch gesellschaftlicher, politischer und wirtschaftlicher Bedeutung.

Ziel dieses Übersichtsartikels ist es, anhand von Beispielen die Spannbreite von Infektionskrankheiten darzustellen, die durch bakterielle zoonotische Erreger ausgelöst werden. Die unterschiedlichen Transmissionswege, epidemischen Potenziale und epidemiologischen Maßzahlen der beispielhaft gewählten Krankheiten sind Herausforderungen für den öffentlichen Gesundheitsdienst, den Tiergesundheitsdienst und die Lebensmittelüberwachung, deren Aufgabe es ist, die Bevölkerung vor diesen Infektionskrankheiten zu schützen.

## Abstract (en)

Bacterial zoonotic pathogens are often the cause of diseases, sometimes with severe outcomes. They are mutually transferable between animals (both wild and domestic) and humans. The transmission paths are very variable and include oral intake via food, respiratory infection via droplets and aerosols, or infections via vectors such as tick bites or rodent contact. Furthermore, the emergence and spread of antibiotic-resistant bacterial pathogens is of paramount public health concern.

The likelihood of further spread is influenced by various factors. These include the increase in international trade, the endangerment of animal habitats, and the increasingly closer contact between humans and wild animals. Additionally, changes in livestock and climate change may also contribute. Therefore, research into zoonoses serves to protect human and animal health and is of particular social, political, and economic importance.

The aim of this review article is to present the range of infectious diseases caused by bacterial zoonotic pathogens in order to provide a better understanding of the important work in public health services, animal health services, and food safety control. The different transmission routes, epidemic potentials, and epidemiological measures of the exemplary selected diseases show the challenges for the public health system to monitor and control the spread of these bacterial pathogens in order to protect the population from disease.

## Einleitung

Bakterielle Zoonosen sind wechselseitig von Tier zu Mensch und von Mensch zu Tier übertragbare Infektionskrankheiten. Sie können als „klassische Zoonose“ mit obligater Tier-Mensch-Übertragung auftreten oder als sogenannte Spillover-Zoonose, bei der im Anschluss an die Tier-zu-Mensch-Übertragung eine Mensch-zu-Mensch-Übertragung erfolgt. Bei klassischen Zoonosen ist die kontinuierliche Kontrolle im Tierreservoir essenziell für den öffentlichen Gesundheitsschutz. Spillover-Zoonosen können gravierende Auswirkungen auf die öffentliche Gesundheit haben, wenn sie sich leicht von Mensch zu Mensch ausbreiten.

Vom Tier ausgehende bakterielle Erreger können beim Menschen schwere, manchmal sogar tödliche Erkrankungen verursachen. In einigen Fällen sind umfangreiche und langwierige Behandlungen notwendig, z. B. beim hämolytisch-urämischen-Syndrom (HUS), das durch enterohämorrhagische *Escherichia coli* (EHEC) ausgelöst wird. In vielen Fällen wird eine Behandlung durch die vermehrte Bildung von Antibiotikaresistenzen erschwert.

Bakterielle Erreger verursachen – als Auslöser von Tierseuchen – in der Landwirtschaft erhebliche ökonomische Schäden und Beeinträchtigungen des Tierwohls. Sie können zu einer erhöhten Sterblichkeit (z. B. Salmonellose, Tularämie), Fehlgeburten (z. B. Q‑Fieber) und Sterilität (z. B. Brucellose) in den Tierbeständen führen. Erhebliche wirtschaftliche Verluste für die landwirtschaftlichen Unternehmen entstehen auch durch Keulung der Bestände oder Handelsbeschränkungen (z. B. bovine Tuberkulose). Die Gesundheit von Gesellschaftstieren (Tiere, von Menschen aus Vergnügen gehalten) wird ebenfalls durch Infektionskrankheiten (z. B. Ornithose bei Vögeln, Leptospirose bei Menschen und Hunden) bedroht. Darüber hinaus können einige bakterielle zoonotische Erreger (z. B. *Bacillus anthracis, Francisella tularensis*) aufgrund ihrer biologischen Eigenschaften für eine böswillige Ausbringung geeignet sein und zu einer Gefahr werden, wenn sie im Rahmen von Bioterrorismus freigesetzt würden.

Zur Sicherstellung der Handlungsfähigkeit der Behörden des Gesundheitsschutzes ist der Labornachweis der meisten zoonotischen Erreger beim Menschen gemäß § 7 Abs. 1 des Infektionsschutzgesetzes (IfSG) dem Gesundheitsamt namentlich meldepflichtig, sofern der Hinweis auf eine akute Infektion besteht. Das Robert Koch-Institut (RKI) erstellt Falldefinitionen für meldepflichtige Infektionskrankheiten zum Zwecke der Infektions-Surveillance [1]. Darauf basierend erfolgt eine Übermittlung der Informationen über die Landesbehörden an das RKI.

Einige bakterielle zoonotische Krankheiten kommen endemisch vor (z. B. Lyme-Borreliose) oder treten sporadisch auf (z. B. Tularämie). Andere Krankheiten können zu Ausbruchsszenarien bei Menschen und Tieren (z. B. Salmonellose, EHEC, Q‑Fieber) führen und stellen eine besondere Herausforderung bei der Ursachenfindung und der Verhinderung von weiteren Erkrankungen dar. Eine der größten durch eine bakterielle Infektionskrankheit ausgelösten Krisen der letzten Jahre war ein durch den Verzehr von rohen Sprossen ausgelöster bakterieller EHEC/HUS-Ausbruch im Jahr 2011 [2].

Dieser Artikel stellt verschiedene bakterielle zoonotische Infektionskrankheiten dar, die exemplarisch das Spektrum der unterschiedlichen Übertragungswege (Lebensmittel, Zeckenstich), Reservoirtiere (landwirtschaftliche Nutztiere, Nagetiere, Gesellschaftstiere) und Problemfelder wie Antibiotikaresistenz abbilden. Ziel ist es, ein besseres Verständnis für die wichtige Arbeit in den öffentlichen Gesundheitsdiensten, den Tiergesundheitsdiensten und der Lebensmittelkontrolle zu vermitteln.

## Infektionskrankheiten durch den Kontakt mit landwirtschaftlichen Nutztieren am Beispiel des Q-Fiebers

Das Q‑Fieber (Query-Fieber) ist eine Zoonose, die durch *Coxiella (C.) burnetii *verursacht wird, ein intrazelluläres, gramnegatives Bakterium mit einem sehr breiten Wirtsspektrum. Die Krankheit wurde erstmals 1937 in Australien beschrieben und kommt, außer in Neuseeland und der Antarktis, weltweit vor [3].

Die Infektion verläuft bei Tieren meistens asymptomatisch, kann aber Reproduktionsstörungen, wie z. B. Aborte, auslösen. Die Erreger werden über Milch, Urin, Kot und insbesondere über Geburtsprodukte (u. a. Nachgeburt und Fruchtwasser) ausgeschieden. Infektionen beim Menschen werden oft durch Kontakt mit Schafen und Ziegen verursacht. Eine Übertragung findet hauptsächlich durch Inhalation kontaminierter Aerosole statt [4]. Dabei sind insbesondere Menschen gefährdet, die beim Geburtsvorgang helfen.

Der Verdacht auf Q‑Fieber kann durch die serologische Untersuchung auf Antikörper gegen *C. burnetii* bestätigt werden. Anhand der Art der Antikörper im zeitlichen Verlauf kann man auf einen akuten oder chronischen Krankheitsverlauf schließen. Beim Menschen löst die Infektion in ca. 50 % der Fälle eine selbstlimitierende grippeähnliche Erkrankung aus (akutes Q‑Fieber). Bei etwa 1–5 % der Patienten kommt es zu einem chronischen Q‑Fieber. Hierbei können u. a. eine Entzündung des Herzens (Endokarditis), infizierte Aneurysmen oder infizierte Gefäßprothesen auftreten [5]. Zwischen der primären Infektion und dem Auftreten von Symptomen können Jahre liegen. Bei Infektionen in der Schwangerschaft besteht ein deutlich erhöhtes Risiko für einen Abort oder eine Frühgeburt.

In der Regel infizieren sich Einzelpersonen. Hierzulande wurden aber auch schon „Superspreading-Events“ beobachtet, bei denen sich eine große Anzahl an Menschen an einem Ort infizierte, wie z. B. bei einem Bauernmarkt in Nordrhein-Westfalen im Jahr 2003 [6].

In Deutschland ist eine Meldepflicht für Q‑Fieber nach dem „Tiergesundheitsrecht“ der Europäischen Union (EU) und im Tiergesundheitsgesetz (TierGesG) geregelt. Infektionen bei Rindern, Schafen und Ziegen sind meldepflichtig nach der Verordnung über meldepflichtige Tierkrankheiten (TKrMeldpflV 1983). Beim Menschen ist der Nachweis von *C. burnetii* gemäß IfSG meldepflichtig.

Zwischen 2012 und 2021 wurden in Deutschland insgesamt 2065 Fälle von Q‑Fieber bei gehaltenen Tieren gemeldet. Dabei entspricht ein Fall einem betroffenen Betrieb, wobei der Fall mehrere infizierte Tiere beinhalten kann. Es wurden 1942 Ausbrüche in Betrieben mit Haltung von Rindern, 108 von Schafen und 13 von Ziegen gemeldet. Die meisten Ausbrüche traten in Bayern (622), Nordrhein-Westfalen (418) und Niedersachsen (329) auf. Dies sind jedoch auch die Bundesländer mit den meisten gehaltenen Tieren.

Im selben Zeitraum wurden 1679 Fälle bei Einzelpersonen an das RKI übermittelt. Männer waren mit 61 % häufiger betroffen als Frauen. Am häufigsten betroffen waren Männer im Alter zwischen 40 und 59 Jahren. Es wurden 1031 Einzelfälle und 57 Ausbrüche mit 648 Fällen übermittelt. Zwei besonders große Ausbrüche wurden im Landkreis Esslingen mit 85 Fällen im Jahr 2016 und im Landkreis Zollernalbkreis mit 64 Fällen im Jahr 2019 identifiziert. Die Anzahl der übermittelten Erkrankungen zeigt, dass es sowohl bei sporadischen Q‑Fieber-Erkrankungen als auch bei Ausbrüchen in Deutschland „starke“ und „schwache“ Jahre gibt, deren genaue Ursache unbekannt ist.

**Figure 1:**
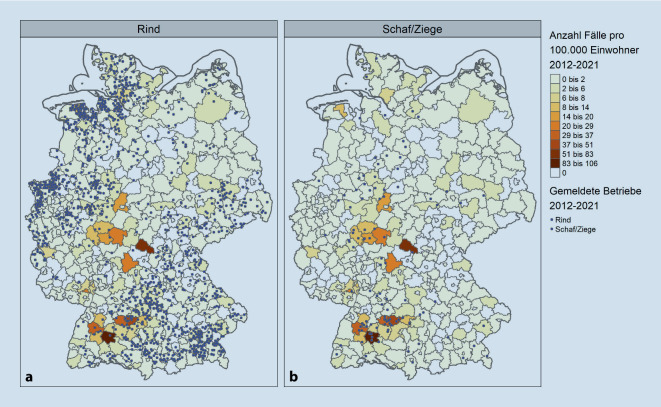

Eine regionale Korrelation zwischen der Anzahl der übermittelten humanen Fälle und den gemeldeten Fällen bei Tieren kann nicht festgestellt werden (Abb. 1). Ein Grund dafür könnte die Einhaltung von Hygienemaßnahmen bei Ausbrüchen in Betrieben sein. Datenartefakte durch ausbleibende Meldungen wären eine alternative Erklärung. Da das Q‑Fieber bei Schafen und Ziegen meist asymptomatisch auftritt und kein aktives Überwachungsprogramm existiert, gibt es hier eine Untererfassung [7]. Auch beim Menschen ist von einer Untererfassung auszugehen, da z. B. die Symptome einer Grippeerkrankung ähneln können und oftmals keine spezifische Diagnostik auf *C. burnetii* erfolgt.

## Lebensmittelübertragene Infektionskrankheiten am Beispiel der Salmonellose

Salmonellosen werden durch Bakterien aus der Gruppe der Salmonellen verursacht und können bei Menschen zu Durchfall, Bauchkrämpfen, Übelkeit und Fieber führen. Salmonellen sind in der Regel bewegliche gramnegative Stäbchen und kommen sowohl bei Menschen als auch bei Tieren hauptsächlich im Verdauungstrakt vor. Umwelteinflüssen gegenüber sind sie relativ robust. Die Bakterien werden in über 2500 Serovare (mittels Tests auf bakterielle Antigene unterscheidbare Variationen) unterteilt. Bei den humanen Erkrankungsfällen wird *Salmonella* (*S.*) Enteritidis gefolgt von *S*. Typhimurium am häufigsten nachgewiesen [8]. Die meisten Infektionsfälle werden durch den Verzehr von rohen oder unzureichend erhitzten Lebensmitteln verursacht.

Die Übertragung von Salmonellen in landwirtschaftlichen Tierbeständen findet unter anderem über Futter, Wildtiere, andere infizierte Tiere oder die Elterntiere, aber auch durch kontaminierte Gegenstände statt. Salmonellen können bei Tieren Krankheitssymptome hervorrufen (z. B. Durchfall) oder Aborte verursachen, jedoch verlaufen die Infektionen häufig unbemerkt. Für die Lebensmittelproduktion sind diese asymptomatischen Verläufe bei Nutztieren problematisch, da die Salmonellen unbemerkt in die weitere Lebensmittelkette gelangen können.

Das Vorkommen von Salmonellen bei Nutztieren, im Tierfutter sowie in Lebensmitteln wird daher systematisch über Monitoringprogramme überwacht [9]. 2003 wurde EU-weit die gesetzliche Grundlage für verstärkte Maßnahmen zur Bekämpfung der Salmonellen in Schwein und Geflügel geschaffen (z. B. für die Impfung von Hühnern) mit dem Ziel, die Erkrankungszahlen beim Menschen zu reduzieren [10]. Studien aus 2004 und 2005 zeigten beispielsweise, dass fast ein Drittel (30,8 %) der Legehennenherden in Europa *Salmonella*-positiv getestet wurde. Nach dem Start nationaler Kontrollprogramme sank die Zahl bis 2014 drastisch auf 0,7 %, stieg danach allerdings wieder an. Im Jahr 2020 wurden 4,0 % *Salmonella*-positive Herden in Europa berichtet. Auch Geflügelfleisch im Einzelhandel war in Deutschland im Jahr 2020 häufig mit Salmonellen belastet (4,6 % der untersuchten Proben).

**Figure 2:**
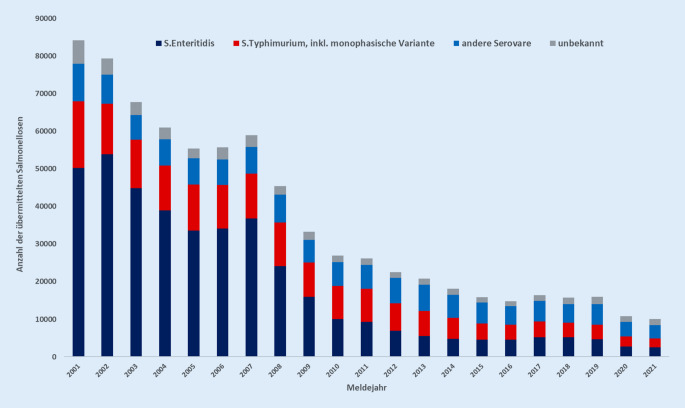

Beim Menschen wird zur Diagnose der Salmonelleninfektion in der Regel eine Stuhlprobe oder bei systemischen Verläufen eine Blutprobe entnommen. Die Probe wird in ein Labor geschickt, wo dann zum Beispiel Bakterienkulturen angelegt werden. Die Anzahl der übermittelten Salmonellosefälle ist in Deutschland im Zeitraum 2001–2014 stark zurückgegangen. Seit 2015 stagnieren die Fallzahlen auf niedrigerem Niveau ohne Trendfortsetzung (Abb. 2). In den Jahren 2020 und 2021 wurde ein weiterer Rückgang der Meldezahlen registriert, wobei ein Einfluss der Maßnahmen zur Eindämmung der COVID-19-Pandemie vermutet wird [11].

Salmonellosen werden in allen Bundesländern registriert, die höchsten Inzidenzen waren in den letzten Jahren in den östlichen Bundesländern zu verzeichnen. In den Sommermonaten treten im Jahresverlauf die meisten Erkrankungsfälle auf [8].

Die Meldeinzidenz bei Kleinkindern unter 5 Jahren ist höher als bei älteren Kindern und Erwachsenen. Männer und Frauen sind ungefähr gleich häufig betroffen. Im Jahr 2020 lag der Anteil der übermittelten Salmonellosefälle, die hospitalisiert werden mussten, in Deutschland bei 26 %.

Immer wieder verursachen Salmonellen große bundeslandübergreifende und internationale Ausbrüche. Die 3 häufigsten Lebensmittelgruppen, die bei Salmonellose-Ausbruchsuntersuchungen in der EU identifiziert werden konnten, sind Eier bzw. Eiprodukte, Schweinefleisch bzw. Schweinefleischprodukte und Backwaren [9]. Insbesondere bei der Untersuchung von Salmonelloseausbrüchen ist eine sektorübergreifende Zusammenarbeit entscheidend. Dies soll hier am Beispiel von 2 Ausbrüchen in Deutschland mit *S.* Muenchen verdeutlicht werden. Beim ersten Ausbruch 2013 wurden 203 und beim zweiten Ausbruch im Jahr 2014 insgesamt 247 Ausbruchsfälle identifiziert. 4 Personen sind mit einer Salmonelleninfektion verstorben, wobei bei einer Patientin die Salmonellose als Todesursache angegeben wurde. Am Ende war diese Ausbruchsuntersuchung erfolgreich darin, die Infektionsvehikel (Rohwürste aus Schweinefleisch) und die wahrscheinliche Ausbruchsquelle (Ferkelaufzuchtbetrieb) zu identifizieren. Dabei lieferten sowohl die Ergebnisse der epidemiologischen Patientenbefragungen und der Mikrobiologie als auch die Rückverfolgung der Lieferwege eine übereinstimmende Evidenz. Durch entsprechende Kontrollmaßnahmen konnten weitere Ausbruchsfälle verhindert werden. Maßgeblich für den Erfolg war die gute Zusammenarbeit der Gesundheitsbehörden mit den Behörden für Lebensmittelsicherheit und Tiergesundheit [12].

## Vektorübertragene Infektionskrankheiten am Beispiel der Lyme-Borreliose

Die Lyme-Borreliose ist die mit Abstand häufigste vektorübertragene Infektionskrankheit in Deutschland mit geschätzt 60.000 bis über 200.000 Fällen pro Jahr. Sie wird in Europa durch wenigstens 5 aus dem mehr als 20 Genospezies umfassenden *Borrelia-*(*B.*)-*burgdorferi-*sensu-lato-Komplex verursacht: Am häufigsten durch *B. afzelii* und *B. garinii*, seltener wird *B. bavariensis, B. burgdorferi *sensu stricto oder *B. spielmanii* nachgewiesen. Die Vektoren sind Schildzecken, für den Menschen in Europa v. a. *Ixodes (I.) ricinus*. Klimawandelbedingt findet sich *I. ricinus* – und damit *B. burgdorferi* – in Europa zunehmend weiter nach Norden verbreitet und auch in höheren Lagen [13]. Die wichtigsten Wirtstiere für *Ixodes* und Erregerreservoire für *Borrelia* sind Mäuse und Vögel, aber auch andere Wirbeltiere wie Reptilien, Igel, Füchse oder Kaninchen.

Die Erkrankungsformen der Lyme-Borreliose werden nach ihrem Manifestationszeitpunkt in „früh lokalisierte“, „früh disseminierte“ und „späte“ Formen unterteilt. Die Hautmanifestation Erythema migrans tritt bei 80–90 % aller Fälle nach einer Inkubationszeit von 3 bis 30 Tage nach Zeckenstich auf. Das Borrelien-Lymphozytom (Schwellung und Verfärbung von Hautpartien) tritt vorwiegend bei Kindern auf.

Nach einer Inkubationszeit von wenigen Wochen kann die frühe Neuroborreliose zumeist als schmerzhafte Entzündung der Rückenmarkshäute und der Spinalnervenwurzeln auftreten. Seltener findet sich eine lymphozytäre Meningitis (v. a. bei Kindern), sehr selten eine Enzephalitis oder Myelitis. Früh disseminierte Formen umfassen u. a. multiple Erythemata migrantia, Lyme-Karditis. Demgegenüber können späte Manifestationen Monate bis Jahre nach dem Stich entstehen. Die Lyme-Arthritis zeigt sich als wiederauftretende, voluminöse Schwellung eines oder weniger großer Gelenke, meist sind Kniegelenke betroffen. Bei der meist an Streckseiten von Extremitäten lokalisierten Acrodermatitis chronica atrophicans (ACA) kommt es zu charakteristischen Veränderungen der Haut und plastischem Hervortreten der Gefäße. Die sehr seltene späte Neuroborreliose zeigt je nach Lokalisation Symptome einer Enzephalitis, Myelitis oder Enzephalomyelitis.

**Figure 3:**
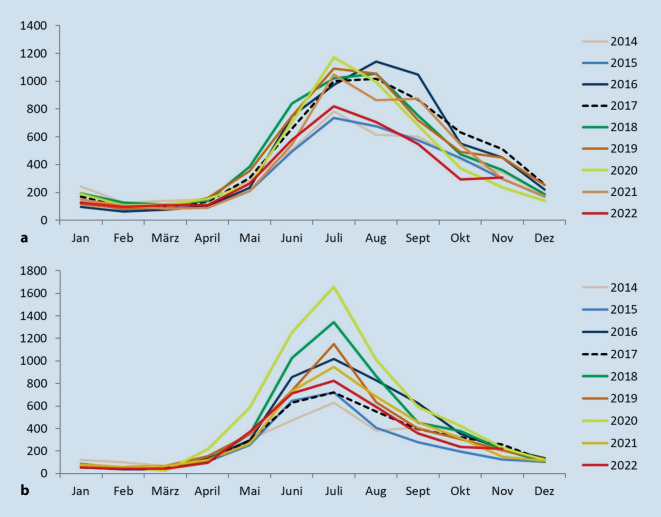

In Europa wird eine Inzidenz von 0,6 in Irland bis 300 in Österreich pro 100.000 Einwohner berichtet, wobei aussagekräftige epidemiologische Studien kaum zur Verfügung stehen [13]. In Deutschland lassen bisher publizierte Daten auf eine Inzidenz von 72 bis > 241 Erkrankungen/100.000 Einwohnern mit starken Jahr-zu-Jahr- und regionalen Schwankungen schließen [14–16]. Dabei zeigt die Lyme-Borreliose ein ausgeprägtes saisonales Muster mit der höchsten monatlichen Fallzahl typischerweise im Monat Juli (Abb. 3). Die Jahr-zu-Jahr-Variabilität der monatlichen Verteilung der Lyme-Borreliose-Fallzahlen ist sehr gering. Eine geringe saisonale Variabilität zeigt sich in der Stärke der Saison (Absolutzahl der Fälle), diese ist aber deutlich weniger ausgeprägt als bei anderen zeckenübertragenen Erkrankungen wie der Frühsommer-Meningoenzephalitis (FSME).

Bevölkerungsrepräsentative deutschlandweite seroepidemiologische Studien an Kindern (KiGGS) und Erwachsenen (DEGS) zeigen, dass die Lyme-Borreliose endemisch ist und dass Kinder < 17 Jahre und Erwachsene > 55 Jahre zu den Risikogruppen zählen. Männer und Jungen haben hierbei ein deutlich erhöhtes Risiko für eine Antikörper-Positivität. Das Gesamtbild deutet eher auf bevölkerungsbezogene Risiken im Bereich Freizeitaktivitäten als im beruflichen Kontext hin. Darüber hinaus zeigen neuere Untersuchungen, dass zwischen 1997–1999 und 2008–2011 pro Zeitraum eines Jahres etwa 0,45 % der Antikörper-negativen Studienteilnehmenden Antikörper-positiv und etwa 1,47 % der Seropositiven wieder negativ werden. Insgesamt ist aber keine signifikante Änderung der Seroprävalenz zwischen den Studienzeiträumen 1997–1999 und 2008–2011 nachweisbar [17–20]. Public-Health-*Handlungsfelder* bei der Lyme-Borreliose sind u. a.:

- regelmäßige Quantifizierung des Einflusses des Klimawandels, z. B. durch regelmäßige bevölkerungsrepräsentative Sero-Surveys,
- Verbesserung der Gesundheitskommunikation, um bestehenden falschen Narrativen bezüglich Diagnose, Therapie und Epidemiologie in der Bevölkerung und bei der ärztlichen Versorgung zu begegnen,
- Aufklärung insbesondere von Outdoor-Zielgruppen (z. B. Pfadfinder, Geocaching-Szene, Waldarbeiter) bzgl. Zeckenstichvermeidung, -erkennung und korrekter Zeckenentfernung sowie Krankheitszeichen der Lyme-Borreliose für einen notwendigen Arztbesuch.

## Nagetierübertragene Infektionskrankheiten am Beispiel der Leptospirose

Die Leptospirose ist eine bakterielle Zoonose, die durch Leptospiren verursacht wird. Leptospiren sind stark bewegliche, korkenzieherartig geformte, obligat aerobe Bakterien. Sie gehören zur Klasse der Spirochäten (schraubenförmige Bakterien). Nach genotypischer Einteilung können derzeit 68 verschiedene Leptospirenarten und bisher insgesamt über 250 Serovare identifiziert werden [21–23].

In der Tierpopulation sind Leptospiren weit verbreitet und können zahlreiche Wild‑, Haus- und Nutztiere wie Hunde, Schafe und Rinder infizieren. Infizierte Nagetiere spielen als Reservoir für die Übertragung auf den Menschen vermutlich die wichtigste Rolle. Diese Hauptwirte erkranken zumeist nicht, scheiden aber den Erreger mit dem Urin mitunter über Jahre aus. Unterschiedliche Leptospirenserovare sind an bestimmte Tierarten adaptiert und jede Tierart kann zugleich Hauptwirt für ein oder mehrere Serovare und Nebenwirt für andere Serovare sein [24].

Die Übertragung auf den Menschen erfolgt meist über direkten und indirekten Kontakt mit dem Urin infizierter Tiere. Die Leptospiren gelangen über die Schleimhäute von Auge, Nase und Mund sowie über kleine Hautverletzungen in den Körper. Nach 7–14 Tagen (manchmal auch 2–30 Tagen) können sehr milde bis sehr schwere Symptome beim Menschen auftreten, die verschiedene Organsysteme betreffen können. Häufig verläuft die Infektion allerdings asymptomatisch oder mit nur leichten unspezifischen Symptomen (vermutlich bei 90 % der Infizierten; [21, 25]).

Aufgrund der sehr variablen Manifestation wird die Leptospirose des Menschen von der Weltgesundheitsorganisation in unterschiedliche klinische Kategorien eingeteilt [23]: 1) grippeähnliche Symptome, 2) Morbus Weil (Gelbsucht, akutes Nierenversagen, Blutungen), 3) Meningitis/Meningoenzephalitis und 4) pulmonale Hämorrhagien mit respiratorischer Insuffizienz.

Von 2001 bis 2021 betrug die durchschnittliche jährliche Inzidenz gemäß IfSG übermittelter Fälle ca. 0,1–0,2 Fälle pro 100.000 Einwohner [21, 26]. Seit 2001 wurden bundesweit jährlich zwischen 35 und 170 Fälle übermittelt – mit ansteigendem Trend (Mittelwerte: 2001-07: 67; 2008-14: 86; 2015-21: 124, Abb. 4). Sero-Surveys in ländlichen Regionen ergaben Antikörper-Seroprävalenzen in der Größenordnung von 4 % [25]. Der Abgleich beider Werte ergibt, dass der Anteil der labordiagnostizierten und durch das Meldesystem erfassten Infektionen im unteren einstelligen Prozentbereich liegt.

**Figure 4:**
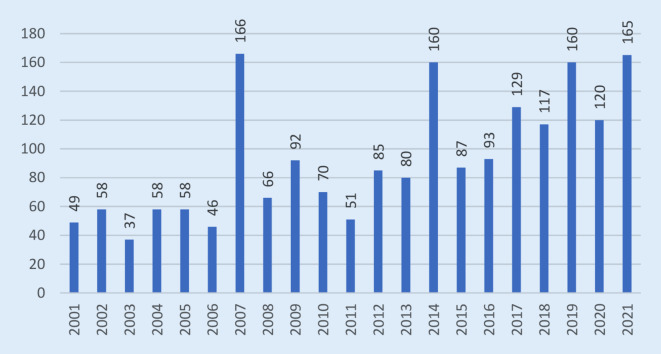

Im Meldesystem erfasste Fälle lassen sich u. a. auf Expositionen im Zusammenhang mit Wassersportaktivitäten, landwirtschaftlichen bzw. Gartenarbeiten und Haltung von Farbratten als Heimtiere sowie mit Auslandsaufenthalten in (sub)tropischen Regionen in Verbindung bringen. Es wurden einzelne größere Ausbrüche in Zusammenhang mit landwirtschaftlicher Exposition oder Wassersportwettbewerben registriert [25, 26].

In den Jahren 2007 und 2014 sind jeweils deutlich mehr Meldefälle erfasst worden als in den benachbarten Jahren. Grund hierfür sind größere Ausbrüche unter Erdbeerpflückenden mit 24 (in Nordrhein-Westfalen; [27]) bzw. 45 Fällen (in Niedersachsen).

Für den Zeitraum 2001 bis 2021 variiert die Meldeinzidenz in den Bundesländern von 0,05 (Thüringen und Sachsen-Anhalt) bis 0,18 (Mecklenburg-Vorpommern) Fällen pro 100.000 Personen und Jahr [24, 28]. Dies sind im Vergleich zu anderen meldepflichtigen Infektionskrankheiten, insbesondere zu den viralen vektorübertragenen Krankheiten wie Hantavirusinfektionen oder FSME, vergleichsweise moderate Schwankungen. Abgesehen von den oben genannten Ausbrüchen wiesen die Infektionen somit eine gering ausgeprägte räumliche Variation auf.

Von den ca. 2000 Meldefällen von 2001–2021 waren 70 % männlich und 30 % weiblich. Über 50 % entfielen in die Altersspanne von 30–59 Jahre, nur 2 % waren Kinder unter 15 Jahren [28]. Die Alters- und Geschlechtsverteilung ist vereinbar mit einem erhöhten Risiko durch berufliche Expositionen in freier Natur, z. B. in der Landwirtschaft, Bauwirtschaft oder Abfallwirtschaft, wo es zu Kontakten mit Nagetierausscheidungen kommen kann [26]. Diesem Muster entsprechen auch die Ausbrüche bei Erdbeerpflückenden.

## Infektionskrankheiten durch Tiere in häuslicher Umgebung am Beispiel der Ornithose

Die Ornithose – auch bekannt als Papageienkrankheit oder Psittakose – wird durch Bakterien der Art *Chlamydia (C.) psittaci* (z. T. als *Chlamydophila psittaci* bezeichnet) verursacht [29–31]. Dabei handelt es sich um unbewegliche, gramnegative Bakterien, die obligat intrazellulär leben. Als Reservoir für *C. psittaci* gelten vor allem Vögel, u. a. Papageien und Sittiche, die als Gesellschaftstiere in häuslicher Umgebung gehalten werden, darüber hinaus aber auch Tauben und Enten. Der in Vogelexkrementen und -sekreten lange überlebensfähige Erreger wird durch Einatmen oder durch unmittelbare Berührung (z. B. Mund-Schnabel-Kontakt, Bisse infizierter Tiere, Umgang mit Federn oder Exkrementen infizierter Tiere) übertragen. Eine Übertragung von Mensch zu Mensch ist sehr selten [30, 32].

Die Inkubationszeit der Ornithose reicht von 1 bis 4 Wochen. Das Krankheitsbild variiert von klinisch unauffälligen Erkrankungen bis zu einer schweren Systemerkrankung. Vorherrschend ist eine fieberhafte Lungenentzündung. Daneben können Kopf‑, Muskel- und Gelenkschmerzen, eine Leber- und Milzvergrößerung sowie Entzündungen des Herzens, des Gehirns, der Leber und der Bindehäute auftreten. Eine Therapie ist mit Antibiotika (Tetrazyklinen, alternativ Makroliden) möglich [30, 31]. Ornithosen treten weltweit vorwiegend sporadisch auf [30]. Darüber hinaus wurden in der internationalen Literatur einzelne Ornithoseausbrüche u. a. im Zusammenhang mit Geflügelschlachtereien beschrieben [33].

Der Verdacht auf Ornithose wird durch die serologische Untersuchung auf spezifische Antikörper bestätigt. In Deutschland ist die Ornithose insgesamt selten. In den Jahren 2001 bis 2021 wurden 426 Ornithosen übermittelt. Mit 9 Erkrankungen wurde in den Jahren 2014 und 2018 die geringste Anzahl und mit 56 Erkrankungen im Jahr 2001 die höchste Anzahl an Erkrankungen übermittelt. Seither ist die Fallzahl auf ein deutlich niedrigeres Niveau gefallen. Angesichts unspezifischer, grippeähnlicher Symptome bei milden Verläufen ist von einer Untererfassung auszugehen [30, 34].

Die geografische Verteilung in Deutschland variiert. Mit 57 Erkrankungen (13 %) wurde von 2001 bis 2021 aus Sachsen die höchste Anzahl an Ornithosen übermittelt, gefolgt von Brandenburg (54), Mecklenburg-Vorpommern (51) und Bayern (49). Die geringste Anzahl an Erkrankungen wurde aus den Stadtstaaten Berlin (4), Bremen (1) und Hamburg (keine) übermittelt.

**Figure 5:**
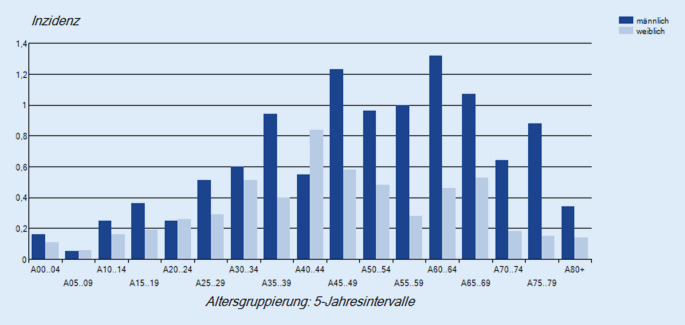

Die erkrankten Personen in Deutschland waren zwischen 1 und 86 Jahren alt (Median 49 Jahre). Unter den Erkrankten waren 148 Frauen (35 %) und 277 Männer (65 %). Die Inzidenz lag in der Altersgruppe der 40- bis 69-Jährigen am höchsten (Abb. 5). Bei 226 Erkrankten (53 %) wurde eine Pneumonie diagnostiziert, bei 5 Erkrankten (1 %) eine Myokarditis. Bei jeweils 2 Erkrankten (0,5 %) lag eine Endokarditis bzw. eine Hepatosplenomegalie vor. Eine Hospitalisierung wurde für 220 Personen (52 %) angegeben, 10 Personen (2 %) sind krankheitsbedingt verstorben.

Kontakt zu potenziell infizierten Vögeln oder ihren Ausscheidungen war bei 177 Erkrankten (42 %) ermittelbar. Unter den Erkrankten mit Angaben zur Vogelexposition bestand häufig Kontakt zu Tauben, Sittichen oder Papageien, z. T. bestanden auch berufliche Expositionen.

Im Zeitraum von 2001 bis 2021 wurden 31 Ausbrüche mit insgesamt 91 Fällen übermittelt. Ein großer Ausbruch mit insgesamt 18 Fällen wurde 2005 in Sachsen-Anhalt im Zusammenhang mit einem Geflügelhandel beschrieben [35].

Zur Prävention einer Ornithose sollten Personen mit Kontakt zu befallenen Vogelbeständen Schutzkleidung, inklusive eines Mund-Nasen-Schutzes, tragen [30]. Bei Auftreten von unklarem Fieber und bekanntem Kontakt zu einzelnen Vögeln oder landwirtschaftlich gehaltenem Geflügel sollte differentialdiagnostisch eine *C.-psittaci*-Infektion berücksichtigt werden.

## Antibiotikaresistenz bei bakteriellen Zoonoseerregern

Neben unterschiedlichen Transmissionswegen und epidemischen Potenzialen der zoonotischen bakteriellen Erreger stellt auch die zunehmende Resistenz einiger Erreger gegenüber Antibiotika (Antimicrobial Resistance – AMR) bei Menschen und Tieren eine Herausforderung für den Gesundheitsschutz dar. Die Zunahme antibiotikaresistenter bakterieller Erreger erschwert oder verhindert adäquate Behandlungen und führt dadurch zu einer hohen Krankheitslast beim Menschen. Im Jahr 2019 konnten weltweit 4,95 Mio. Todesfälle mit resistenten Bakterien in Verbindung gebracht werden, wobei mindestens 1,27 Mio. Todesfälle direkt auf die Antibiotikaresistenz zurückzuführen waren [36]. Davon ist ein Anteil mit hoher Wahrscheinlichkeit auf eine zoonotische Übertragung von resistenten Bakterien zwischen Tieren, kontaminierten Lebensmitteln und Menschen zurückzuführen, wie z. B. bei ESBL-/AmpC-/carbapenemase-produzierenden *E. coli*, dem livestock-assoziierten Methicillin-resistenten *Staphylococcus aureus* (LA-MRSA) und bei lebensmittelübertragenen Erregern wie *Salmonella* und *Campylobacter *[37].

**Figure 6:**
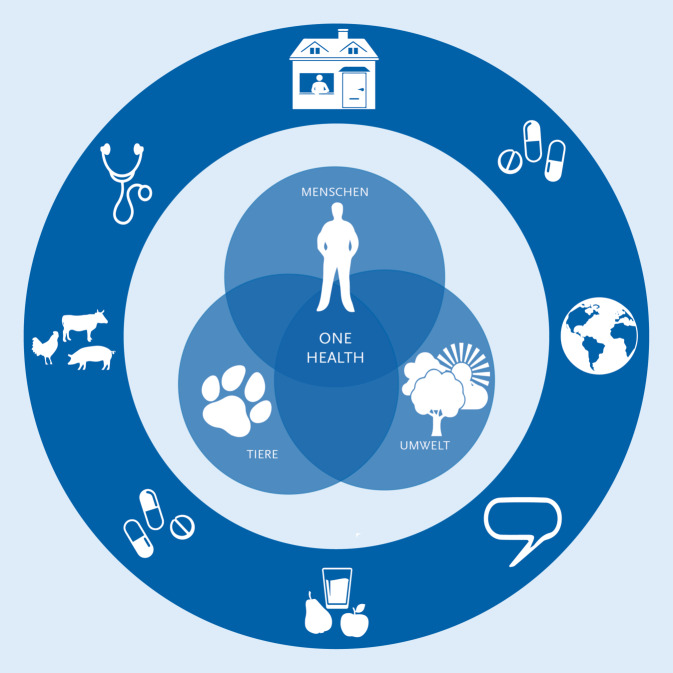

Der One-Health-Ansatz als Konzept zur Förderung der interdisziplinären Zusammenarbeit insbesondere zwischen Humanmedizin, Veterinärmedizin und Umweltwissenschaften hat innerhalb der Antibiotikaresistenz-Thematik das Ziel, die Antibiotikaabgabe und das Vorkommen von antibiotikaresistenten Bakterien interdisziplinär und sektorübergreifend zu untersuchen (Abb. 6; [38]). Dieser Ansatz ist auch Teil der Deutschen Antibiotika-Resistenzstrategie der Bundesregierung (DART 2020; [39]) und findet sich in den Zielstellungen vieler Organisationen der Vereinten Nationen [40]. Im internationalen Kontext erstellt die Europäische Behörde für Lebensmittelsicherheit (EFSA) in Zusammenarbeit mit dem Europäischen Zentrum für die Prävention und die Kontrolle von Krankheiten (ECDC) jährlich eine vergleichende Analyse von AMR-Monitoring- und Überwachungsdaten für *Salmonella*-, *Campylobacter*- und *E.-coli*-Isolate in den wichtigsten lebensmittelproduzierenden Tierpopulationen, in Lebensmittelproben und beim Menschen [41].

Um das Vorkommen von Antibiotikaresistenzen bei Menschen, Lebensmitteln und Tieren in Deutschland zu vergleichen, haben das RKI und das Bundesinstitut für Risikobewertung (BfR) eine Analyse im One-Health-Kontext durchgeführt. Dabei wurden die etablierten Systeme der Antibiotika-Resistenz-Surveillance (ARS; [42]), des Zoonosen-Monitorings [43] und des Resistenzmonitorings für Tierpathogene (GE*RM*-Vet; [44]) genutzt. In einem ersten Schritt wurden Resistenzdaten von *E. coli* untersucht. In dieser Studie wurden 333.496 *E.-coli*-Isolate aus 41 verschiedenen Humanpopulationen (ambulant und von Normal- und Intensivstationen) und Tierpopulationen (aus Erzeugerbetrieben, Schlachthöfen und dem Einzelhandel) eingeschlossen. Die Resistenzanteile (in %) für 4 Antibiotika (Ampicillin, Cefotaxim, Ciprofloxacin und Gentamicin) wurden zwischen bakteriellen Isolaten von Menschen und Tieren verglichen [45]. Insgesamt waren die Resistenzanteile bei Ampicillin am höchsten (1–70 %), gefolgt von Ciprofloxacin (0–36 %), Cefotaxim (0–16 %) und Gentamicin (0–15 %). Die Resistenzanteile für Ampicillin variierten bei menschlichen klinischen Isolaten von 43 % bis 55 %, bei gesunden lebensmittelliefernden Tieren und Lebensmitteln von 1 % bis 70 % und bei klinischen Tierisolaten von 16 % bis 64 %. Darüber hinaus konnte die Vergleichsanalyse mögliche Übertragungswege zwischen unterschiedlichen Menschen- und Tierpopulationen anhand ähnlicher Resistenzmuster identifizieren [45]. Diese Übertragungswege zeigen sich in der Analyse insbesondere bei der Mensch-zu-Mensch-Übertragung und dem Verzehr von rohem Fleisch, wie z. B. rohem Schweinefleisch [46].

Als Herausforderung für den Vergleich der Resistenzsituation zwischen Human- und Veterinärmedizin stellen sich die Unterschiede bei den routinemäßig getesteten Wirkstoffen zwischen den Sektoren dar. Darüber hinaus stellen die Einführung unterschiedlicher klinischer und epidemiologischer Grenzwerte sowie die Nutzung unterschiedlicher Normensysteme wie EUCAST^1^ und CLSI^2^ Barrieren dar. Eine sektorübergreifende Harmonisierung wäre daher wichtig, um die integrierten Analysen zu optimieren. Langfristig sollte eine gemeinsame webbasierte Plattform zu einem besseren Verständnis für das Problemfeld der Antibiotikaresistenz und zu Lösungsmöglichkeiten im Bereich der öffentlichen Gesundheit führen.

## Fazit

Die in diesem Artikel dargestellten epidemiologischen Situationen der beispielhaft gewählten Themengebiete Salmonellose, Q‑Fieber, Lyme-Borreliose, Leptospirose, Ornithose und Antibiotikaresistenz zeigen, dass es große Unterschiede bei den Reservoiren, den Transmissionswegen, der Inzidenz und den notwendigen Maßnahmen zur Verhinderung von bakteriellen Infektionen im Bereich des öffentlichen Gesundheitsschutzes gibt. Lyme-Borreliose und Salmonellose, aber auch Campylobacteriose verursachen aufgrund ihres häufigen Vorkommens eine besondere Krankheitslast [47]. Die Trends bei der Inzidenz zu detektieren, ist bei diesen Krankheiten besonders wichtig. Andere Krankheiten wie das Q‑Fieber oder die Ornithose kommen eher selten vor und finden daher selten Eingang in die routinemäßige Patientendiagnostik, was zu einer Untererfassung führt.

Veränderungen in unserer Lebensweise und engere Kontakte mit Tieren haben das Auftreten einiger bakterieller Infektionen verstärkt, verändert oder neu entstehen lassen. Bakterielle Zoonosen gehören zu den Infektionskrankheiten, die insbesondere wieder auftreten können, wenn sie als „getilgt“ oder „unter Kontrolle“ gelten (z. B. bovine Tuberkulose). Auf der anderen Seite haben Verbesserungen in der Surveillance und in der Diagnostik dazu geführt, dass bakterielle Zoonosen zunehmend besser kontrolliert werden.

Besonders exponiert gegenüber zoonotischen Erregern sind alle Personen mit Tierkontakten sowie Beschäftigte aus der Lebensmittelproduktion (z. B. Schlachthöfe, Milch- und Geflügelbetriebe). Sie sind einem erhöhten Infektionsrisiko ausgesetzt. Darüber hinaus haben Personen, die Risikolebensmittel roh verzehren, ein erhöhtes Risiko für u. a. Yersiniose, EHEC-Infektion oder Salmonellose. Menschen mit geschwächtem Immunsystem sind besonders gefährdet, einen schweren Krankheitsverlauf bei Infektionen mit bestimmten Zoonoseerregern zu erleiden. Darüber hinaus kann es bei Schwangeren zur Infektion des Ungeborenen kommen (z. B. Listeriose). Die Behörden der Lebensmittelsicherheit geben detaillierte Verzehrempfehlungen für unterschiedliche Gruppen, um Infektionen zu vermeiden.

Die vielfältigen Transmissionswege, die epidemischen Potenziale und die unterschiedlichen epidemiologischen Maßzahlen bei bakteriellen Zoonosen stellen das öffentliche Gesundheitswesen vor große Herausforderungen bei der Überwachung und Eindämmung der Krankheitsausbreitung zum Schutz der Bevölkerung. Viele bakteriologische, infektiologische und epidemiologische Aspekte dieser Erkrankungen sind immer noch unbekannt. Deshalb sollten bakterielle Zoonosen auch in den nächsten Jahren Gegenstand von Public-Health-Bemühungen und Forschungsprojekten im multisektoriellen (One-Health‑)Ansatz sein [48].

## Footnotes

1 EUCAST: European Committee on Antimicrobial Susceptibility Testing.

2 CLSI: Clinical and Laboratory Standards Institute.
